# GLP-1 Receptor Agonist Use in Cancer Survivors—Challenges and Opportunities: A Narrative Review

**DOI:** 10.3390/cancers18162714

**Published:** 2026-08-21

**Authors:** Tanya Agurs-Collins, Edward R. Sauter

**Affiliations:** 1 Division of Cancer Control and Population Sciences, National Cancer Institute, National Institutes of Health, 9609 Medical Center Drive, Rockville, MD 20850, USA; 2 Division of Cancer Prevention, National Cancer Institute, National Institutes of Health, 9609 Medical Center Drive, Rockville, MD 20850, USA

**Keywords:** incretin mimetics, GLP-1 medicines, cancer, cancer survivors

## Abstract

Glucagon-like peptide (GLP)-1 receptor agonists (GLP-1RAs) (including medications that contain GLP-1 and other RAs), hereafter referred to as GLP-1 medicines, have significantly advanced the management of metabolic disease and weight management. Obesity is highly prevalent among cancer survivors at the time of diagnosis, and cancer treatments are associated with subsequent changes in weight, either gain or loss. Research has shown that obesity can adversely affect cancer treatment outcomes and survivorship, leading researchers to investigate the potential integration of GLP-1 medicines into oncology care. There is growing interest in understanding the effects of GLP-1 medicines on weight loss and cancer-related outcomes among individuals with obesity who are taking anticancer therapies, as well as individuals treated for cancer in the past who are long-term survivors. This review examines the literature to characterize what is known about GLP-1 medicine use among cancer survivors. The current literature, though limited and largely retrospective, suggests that GLP-1 medicines may contribute to weight loss among cancer survivors, with outcomes varying by cancer stage and type of anticancer therapy. It is important for healthcare providers to consider the potential negative impact of these GLP-1 medications on their patients, especially those with cancer cachexia or sarcopenic obesity, as well as those undergoing anticancer treatments that often lead to weight loss. While there is preclinical evidence suggesting that there are weight-loss independent effects that may be beneficial to individuals with cancer, use of GLP-1 medicines in patients of normal weight for reasons other than treatment of type 2 diabetes should be initiated with caution. On the positive side, these agents may help mitigate therapy-related toxicities, such as cardiotoxicity, with the potential to enhance survivorship outcomes. Unfortunately, there is a notable lack of well-designed randomized controlled trials evaluating the effects of GLP-1 medicines on weight loss, clinical endpoints, and cancer-related outcomes. Advancing the field requires a focus on elucidating the interactions between GLP-1 medicines and anticancer therapies, particularly their impact on treatment efficacy, nutritional status, and overall patient outcomes, both during treatment and in the long term.

## Simple Summary

Approximately 31.4% of cancer survivors aged 20 years and older have obesity, a condition that can negatively affect cancer treatment outcomes and overall survivorship. Addressing and preventing obesity may reduce the risk of cancer recurrence and improve long-term health outcomes. As a result, researchers are increasingly exploring the integration of glucagon-like peptide (GLP)-1 receptor agonists (GLP-1RAs), hereafter referred to as GLP-1 medicines, into oncology care. However, cancer survivors face unique challenges both during and after treatment, which may impact the use of GLP-1 medicines. This review found limited research examining GLP-1 medicine use in individuals both during and after cancer treatment. Existing studies suggest that GLP-1 medicines may lead to weight loss, though the amount of weight lost is influenced by many factors, including the anticancer therapies used. Moreover, healthcare providers prescribing GLP-1 medicines during cancer treatment need to consider the potential negative impact of these medications on cancer patients, especially those who may have or may develop cancer cachexia or sarcopenic obesity, as well as those undergoing treatments that often lead to weight loss. On the other hand, preliminary findings indicate that GLP-1 medicines may help reduce certain treatment-related toxicities, such as cardiotoxicity. Well-designed studies that comprehensively evaluate potential interactions between GLP-1 medicines and anticancer treatments, particularly their effects on weight, cancer-related outcomes, and overall patient health, are lacking and are urgently needed.

## 1. Introduction

According to the National Cancer Institute [1], in 2023 approximately 31.4% of cancer survivors aged 20 years or older had obesity. Obesity prevalence increased more rapidly from 1997 to 2014 among cancer survivors vs. the general population, with colorectal, breast and prostate cancer survivors all having an increase in obesity risk during the period [2]. Cancer survivors include individuals undergoing active treatment, as well as those who have completed treatment. Many studies evaluating survivors include both groups. 

### 1.1. Do Obese Patients Have Better or Worse Outcomes than Those Who Are Not Obese?

A meta-analysis involving over 6 million participants observed that obesity was associated with increased overall and cancer-specific mortality compared to non-obese survivors, especially among individuals with breast, colon and uterine cancer. On the other hand, obese individuals with renal cell carcinoma, lung cancer and melanoma had better survival than non-obese patients [3]. The “obesity paradox” for some cancers, in which obese patients were found to have better outcomes than those who are non-obese, remains under debate. Explanations posited to explain the seeming paradox include: (1) weaknesses in the method for assessing obesity, (2) inadequately accounting for the heterogeneity of the obese vs. non-obese groups other than cancer diagnosis, and (3) possible true biologic benefits of obesity. Regarding the first explanation, one weakness is the fact that body mass index (BMI) is a relatively poor measure of adiposity. Other measures of obesity, whether clinical (such as waist circumference and waist to hip ratio), or imaging (such as computerized tomography or magnetic resonance imaging) studies have failed to demonstrate that obese cancer survivors fare as well as their non-obese counterparts [4]. Regarding the second explanation, epidemiologic investigations are often subject to confounding and bias of various kinds. For example, BMI tends to be higher in non-smokers than in smokers, the latter of whom often have unintended weight loss due to their disease and/or comorbidities. Moreover, unintentional weight loss in the absence of smoking, which is observed with some cancer treatments, may be associated with cancer cachexia and poorer outcomes. On the other hand, there is evidence that in some tumor types (including renal cell cancer), obese individuals, on average, appear to have less aggressive disease. Moreover, some studies report that obese individuals respond better to immune checkpoint inhibitor (PD-1/PD-L1) treatment [3].

### 1.2. Cancer-Specific Negative Effects of Obesity

Obesity impacts the efficacy of screening for both colorectal and breast cancer, which could delay or prevent detection of disease recurrence or the full measure of disease extent. A colonoscopy may be delayed or skipped due to participant concerns over weight stigma, or the equipment may not be able to accommodate their weight [5]. Mammograms may be less effective in obese women, as excess breast tissue can lower image quality [6]. Obesity negatively impacts cancer treatment. Surgical procedures for cancer increase operative times and complication risk [7]. The risk of radiation-induced toxicity is increased with obesity, including higher risk of radiation dermatitis [8], and increased risk of lymphedema [9] after nodal removal. The pharmacokinetics of some chemotherapeutic agents are altered by obesity, especially if the agent is fat soluble, with the possibility of under- or over-dosing. On the other hand, as already mentioned, there is evidence that obese individuals respond better to immune checkpoint inhibitors [3].

### 1.3. What Is the Scientific Rationale to Expect GLP-1 Medicines to Favorably (Or Unfavorably) Impact Cancer Survivors?

Incretin mimetics, which stimulate insulin release and reduce glucagon secretion, were initially approved for control of type 2 diabetes mellitus (T2DM) and more recently have been approved for use in individuals with overweight and obesity without T2DM. The early mimetics were single agonist glucagon-like peptide (GLP)-1 receptor agonists (GLP-1RAs). Newer mimetics, such as the dual agonist tirzepatide, which contains a second mimetic, glucose-dependent insulinotropic polypeptide (GIP), as well triple and quadruple agonists, are now approved (in the case of tirzepatide) or under study. For simplicity, hereafter we refer to all mimetics that contain GLP-1RAs as GLP-1 medicines. The short-term benefits of these agonists for glucose control and weight loss are well documented. Studies suggest that individuals with overweight or obesity, with or without T2DM, who are treated with GLP-1 medicines, may have a reduced incidence of some obesity-related cancers [10,11]. Given the substantial weight loss often associated with GLP-1 therapy, there is growing interest in evaluating the role of these agents among cancer survivors with overweight or obesity who are undergoing as well as those who have completed cancer treatment. Many cancer survivors have overweight or obesity at the time of diagnosis, and several cancer treatments, such as endocrine therapy, are associated with subsequent weight gain, while other treatments, such as cisplatin, can lead to weight loss, as well as muscle wasting and atrophy [12,13]. The magnitude of weight change varies according to clinical characteristics, cancer type, and treatment modality [14,15]. However, research examining GLP-1 medicine use among cancer survivors is still emerging. Understanding if and when GLP-1 medicines should be used, especially among survivors under active cancer treatment, requires considering the impact of therapy on an individual’s weight and frailty. Further, it is important to understand whether GLP-1 medicine-associated weight loss can influence cancer recurrence, treatment outcomes, and survival. This narrative review identified emerging clinical studies on GLP-1 medicine use among cancer survivors with obesity, with particular attention to nutritional considerations and potential treatment-related safety concerns.

#### 1.3.1. Effects Related to Weight Gain or Loss

GLP-1 medicines are approved for both obese and non-obese individuals with T2DM and for obese (BMI ≥ 30 kg/m^2^) or overweight individuals (BMI 27–29.9) with at least one weight-related comorbidity. Weight gain, primarily due to increased fat mass, is often observed during cancer treatment among individuals with commonly diagnosed cancers such as those of the breast [16], prostate and colorectum [2]. This obesity, which is often associated with the loss of muscle mass (so-called sarcopenic obesity), negatively impacts mobility and other bodily functions. Moreover, obesity is associated with an increased risk of cancer recurrence, disease-specific mortality, overall mortality, comorbid conditions, and poor quality of life [3,17].

The Phase III SURMOUNT-5 trial enrolled individuals with overweight or obesity without T2DM. The study compared the two most commonly used single (semaglutide) and dual agonist (tirzepatide) GLP-1 medicines for 72 weeks, observing a mean reduction in body weight of 20.2% with tirzepatide compared with 13.7% among semaglutide users [18]. In the tirzepatide arm, 81.6% of individuals and in the semaglutide arms, 60.5% of the individuals lost at least 10% of their body weight. Unfortunately, these encouraging clinical trial results are often not replicated in real-world data. A real-world study conducted in 2019–2024 found that 24.5% of individuals stayed on GLP-1 medicines, including exenatide, liraglutide, semaglutide and tirzepatide, for ≥a year and are therefore at risk of weight regain [19]. More recent findings suggest that 63% of individuals who started on semaglutide or tirzepatide in 2023 took them for over a year. The report speculated that more individuals were staying on the medications for obesity as product shortages ease, insurance coverage expands and physicians better manage side effects [20]. There may be a way to mitigate the lack of persistence in agent use among some individuals, in that there is early evidence that reduced-frequency maintenance dosing of GLP-1 medicines may preserve weight loss [21]. As we await broader population data, there is increasing interest in evaluating weight loss outcomes and identifying optimal dosing strategies for weight management in cancer survivors.

#### 1.3.2. Weight-Loss Independent Effects

There is evidence that GLP-1 receptor signaling has intrinsic anti-inflammatory actions that are not dependent on weight loss [22]. Inflammation is a known driver of cancer development and progression. Moreover, by increasing insulin release, GLP-1 medicines also decrease insulin resistance [23]. Metabolic dysfunction-associated steatotic liver disease (MASLD) significantly increases the risk of liver and various extrahepatic cancers [24]. GLP-1 medicines have been observed to improve liver parameters (liver function tests) in patients with MASLD. Preclinical evidence also supports the role of GLP-1-based medicines in immune reprogramming. In a murine model of pancreatic adenocarcinoma, semaglutide given prior to pancreatic cancer organoid implantation decreased obesity-associated desmoplasia (including collagen deposition), and increased T-lymphocyte infiltration within tumors [25]. Similarly, retatrutide, a triple-agonist incretin mimetic, reduced tumor engraftment, delayed tumor onset, and markedly blunted tumor progression. These antitumor effects persisted after drug withdrawal despite substantial weight regain. This was accompanied by persistent changes in leptin and interleukin-6 (IL-6) [26]. Also, GLP-1 medicines improved liver parameters (improved liver function tests) in MASLD independent of weight loss [27]. The beneficial effects of GLP-1 medicines are important since inflammation [28], insulin resistance [29], and immune reprogramming leading to increased T-cell infiltration [30] have been associated with poor outcomes in individuals with cancer.

The overwhelming bulk of data addressing the mechanisms by which GLP-1 medicines work is from preclinical investigations. While preclinical study results are not always borne out in clinical trials, they provide important insights which can help guide the development of human studies. As such, below we provide examples of preclinical studies supporting the weight-loss independent efforts of GLP-1 medicines on obesity-related cancers:

Breast: In preclinical models of breast cancer, semaglutide (GLP-1 medicine) was analyzed to determine its effect on tumor growth and progression in female BALB/C wild-type premenopausal mice, aged eight to ten weeks, and two in vitro breast cancer cell lines of murine and human origin. Semagltide demonstrated antitumor immunity effects, by decelerating breast cancer growth and progression [31].

Thyroid: There is also evidence that GLP-1 medicines may have direct antitumor effects. In preclinical thyroid cancer models, semaglutide suppressed tumor growth by shifting macrophage polarization from an M2 to a M1 phenotype, which is consistent with the observed tumor-suppressive effect [32]. The influence of semaglutide on macrophages was shown to be direct and to occur through suppression of proxisome Proliferator-Activated Receptor (PPAR).

Endometrial: The efficacy of tirzepatide in a transgenic mouse model of endometrial cancer was assessed [33]. Four weeks of tirzepatide treatment significantly decreased body mass and tumor masses in obese and non-obese mice. Expression of the proliferation marker Ki67 and the anti-apoptotic marker Bcl-xL were lower with tirzepatide treatment.

Prostate Cancer: Human prostate cancer cell lines (CWR-R1, 22Rv1, MDA-PCa-2B, LAPC4) and prostate cancer RNA-seq datasets were analyzed to understand the systemic effects of semaglutide and potential impacts on prostate cancer cells [34]. Results reveal that GLP-1 medicines blocked oncogenic signaling pathways and growth alone and in combination with enzalutamide. Because GLP-1 medicines delay gastric emptying, there has been concern that they may decrease overall drug exposure to drugs such as enzalutamide. Calvarysky et al. evaluated this concern, concluding that while GLP-1 medicines could increase, decrease, or have no effect on overall drug exposure, it was not considered clinically significant [35].

Pancreas: Primary tumor specimens from non-diabetic patients, two human pancreatic cancer cell lines (MIA PaCa-2 and PANC-1) and five-week-old male athymic nude mice were evaluated to determine the impact of a GLP-1 medicine on pancreatic cancer cells [36]. The results suggest that GLP-1R activation by liraglutide inhibited tumorigenicity and metastasis both in vitro and in vivo, mediated by the inhibition of PI3K/Akt signaling pathways.

Ovarian: In two ovarian cancer cell lines (SKOV-3 and CAOV-3), the effects of GLP-1 and Exendin-4 on migration, apoptosis and metalloproteinase production were examined [37]. Study results suggest that Exendin-4 suppresses ovarian cancer migration and induces apoptosis by the activation of GLP-1R in human ovarian cancer cells, indicating potential additive or synergic effects of incretin-chemotherapy treatment. Also observed were anti-inflammatory effects, partially mediated by modulation of the NF-κB signaling pathway.

Overall, current research suggests that GLP-1 medicines may influence cancer biology by improving metabolic health and reducing systemic inflammation, which may in turn decrease cancer cell migration, proliferation, and tumor growth. However, human studies investigating these associations remain limited. GLP-1 medicines have been shown to be cardioprotective, and tirzepatide has also been observed to decrease bone loss [38]. The risk of hypoglycemia among individuals taking GLP-1 medicines of normal level glucose is considered low [38]. A meta-analysis observed a reduction in the inflammatory cytokine C-reactive protein in patients with T2DM receiving semaglutide [39]. As already mentioned, while both semaglutide and tirzepatide are FDA approved for use in T2DM patients who are of normal weight, these agents are not approved for non-diabetics who are not overweight or obese. As these agents can cause side effects, they should be used with caution in cancer survivors who are of normal weight, especially during active cancer treatment. Rigorous, well-designed research is needed to clarify both the direct and indirect effects of these agents on weight loss, clinical endpoints, and cancer-related outcomes in both obese and non-obese individuals.

## 2. Materials and Methods

**Figure 1:**
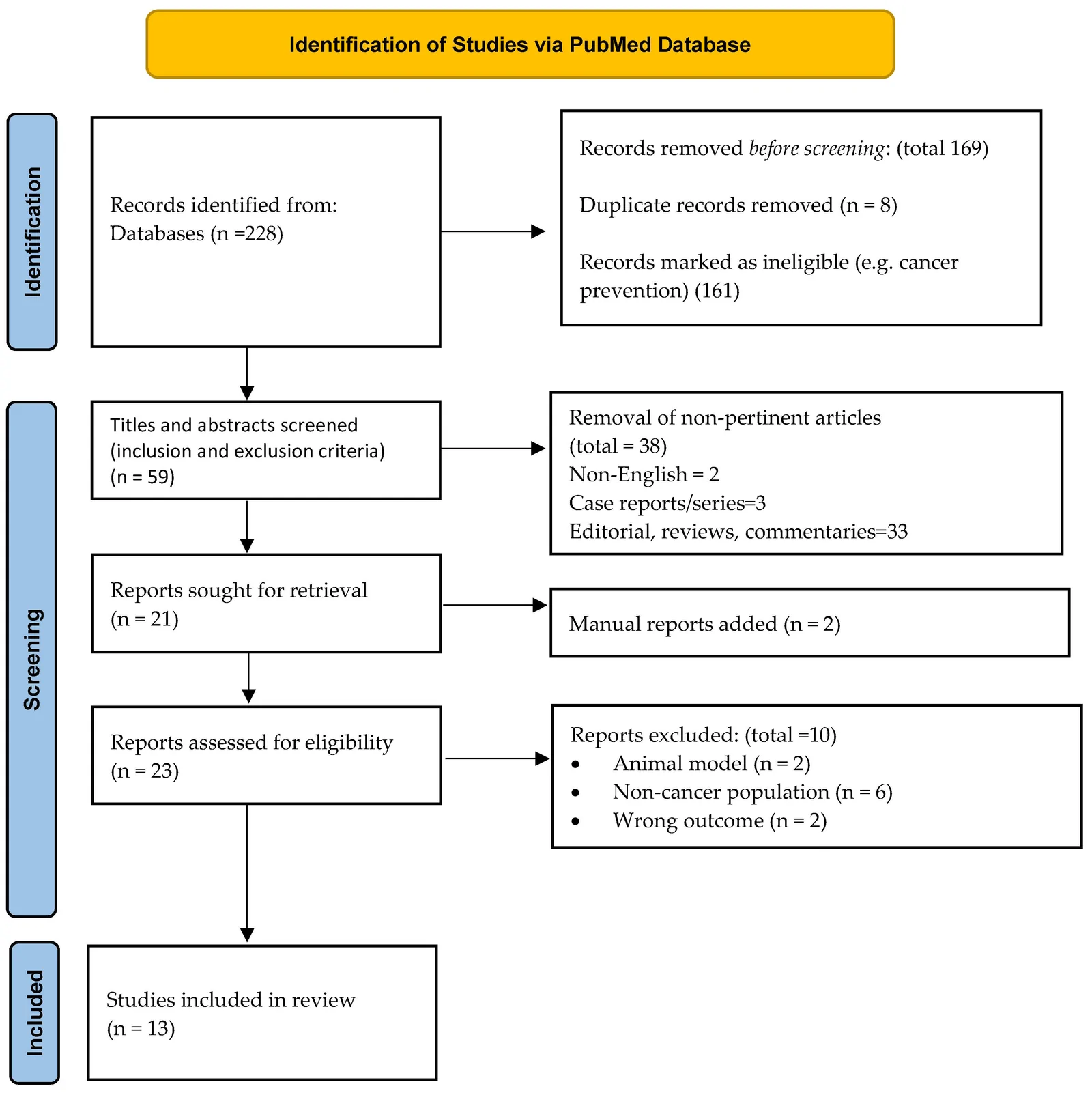
Flowchart of the selected studies.

We performed a narrative review of the literature reported in PubMed/MEDLINE between 2016 and April 2026, using the following combinations of key terms: cancer treatment, cancer survivor, breast cancer, GLP-1, and incretin mimetic. For screening and review of articles, we used a two-stage review process for study selection: title and abstract screening and full-text screening. Two reviewers (TAC, ERS) independently rescreened and reviewed the articles. Inclusion criteria included the following: (1) written in English; (2) full text available; (3) any country; (4) cancer survivor; (5) GLP-1 or incretin mimetic; (6) cancer treatment; (7) an original empirical study published in a peer-reviewed journal or published abstracts. Articles were excluded based on the following criteria: (1) cancer risk; and (2) retraction, editorial, commentaries, reviews, case reports, or case series. Additional references were identified through manual review of the references in the key articles. Two hundred twenty-eight studies were identified, of which 13 were relevant for this review (see the flow diagram in Figure 1). The studies are discussed below in two sections: (1) GLP-1 medicines among individuals with cancer and (2) GLP-1 medicines and cancer toxicity.

## 3. GLP-1 Medicines Among Individuals with Cancer

A substantial proportion of cancer survivors present with obesity at the time of diagnosis and may experience additional weight gain or loss related to treatment type. However, evidence evaluating whether GLP-1 medicine therapy as an effective weight management strategy for specific subgroups of cancer survivors with obesity remains limited. Two retrospective studies published in 2025 and 2026 examined GLP-1 medicines among cancer survivors and reported weight outcomes [40,41]. A recent study among 1022 breast cancer survivors (stages I–II; 79% with T2DM) receiving a GLP-1 medicine for at least 3 months after diagnosis evaluated treatment patterns, weight loss, and outcomes [41]. Approximately 53% received chemotherapy, and 66% endocrine therapy. The investigators found that among GLP-1 medicine users, the median weight change at 12 months was −2.6% (−27.8% to 11.5%), and that endocrine therapy and invasive breast cancer were associated with weight gain and loss, respectively. Because the study focused on individuals with T2DM, who generally lose less weight than those lacking T2DM, the findings may not be generalizable to the entire breast cancer survivor population.

Another retrospective study examined weight data before and during treatment with GLP-1 medicines among patients with breast cancer [40]. Although weight loss at 12 months was 5% for the cohort, the median weight change was lower for patients using concurrent endocrine therapy compared with non-concurrent users at −3% (range, −18% to 9%) versus −5% (range, −22% to 4%), respectively [40]. It should be noted that there is variability in the amount of weight loss with GLP-1 medicines. This variability can depend on presence/absence of T2DM, whether the individual has had prior metabolic bariatric surgery, menopausal status for women, and the specific GLP-1 medicine and duration on the agent. The variability increases among cancer survivors who are under active treatment.

Initial investigations of the impact of GLP-1 medicine use on cancer survivorship generally evaluated individuals receiving GLP-1 medicines or controls who subsequently developed cancer. We identified six studies [42,43,44,45,46,47] among cancer survivors taking GLP-1 medicines and their impact on mortality and/or overall survival. Data from the California Health Data Warehouse were evaluated for the impact of GLP-1 medicine use on mortality among individuals with colon cancer [43]. Study results found that 5-year all-cause mortality was lower (15.5% vs. 37.1%, OR = 0.38, 95% CI: 0.21–0.64) among users compared with non-users [43] and the benefit persisted after adjusting for additional confounders. Similar results were reported in a retrospective cohort study of female patients diagnosed with ovarian cancer [47]. This study found that GLP-1 medicine users had significantly lower 5-year all-cause mortality compared with non-users (7.94% vs. 19.71%; hazard ratio [HR], 0.45; 95% CI: 0.35–0.59; log-rank *p* < 0.001) [47]. However, the incomplete matching on stage and small sample size for some subgroup analyses limits the generalizability of the findings. Another report involving 1502 individuals with colorectal cancer and obesity supported the beneficial effects of GLP-1 medicines on mortality [44]. GLP-1 medicine use compared with non-use was associated with a lower risk of overall mortality (HR = 0.58, 95%CI: 0.45–0.76, *p* < 0.001) [44]. An interesting study evaluated patients with T2DM and active cancer who were receiving GLP-1 medicines compared with metformin. Among patients initiating systemic cancer therapy, GLP-1 medicine use within 3 months of treatment initiation was associated with significantly reduced all-cause mortality compared with metformin use alone (HR = 0.86, 95% CI: 0.78–0.99, *p* < 0.0268) [46]. However, when stratified by BMI, obese patients receiving GLP-1 medicines demonstrated improved survival, but the association did not reach statistical significance [46]. Fawzy et al. [45] found that GLP-1 medicine use among adult patients with neuroendocrine neoplasms was associated with a 44.3% reduction in mortality risk among patients with T2DM or obesity versus non-users. Unfortunately, the study was unable to assess GLP-1 medicine on tumor stage and grade as well as time from diagnosis to initiation of treatment, which limits the interpretation of the findings. Similar findings were reported in colon cancer survivors when comparing GLP-1 medicine users and non-users [42]. The investigators observed improved all-cause mortality (HR = 0.46, 95%CI: 0.40–0.53, *p* < 0.001). These findings suggest that GLP-1 medicines may mitigate risk through pleiotropic effects across multiple organ systems in adults [41]. For example, others reported beneficial effects of GLP-1 medicine use on chronic diseases such as cardiovascular (CV) and renal disease on all-cause mortality among individuals with T2DM [48], suggesting broad organ-specific effects that are independent of weight loss. Taken together, these studies suggest GLP-1 medicines may improve overall survival but not disease-specific mortality. However, interpretation of these findings is limited by the potential for bias inherent in retrospective study designs, as well as heterogeneity in patient populations (including cancer stage, menopausal status, and T2DM status), limited sample sizes, and inadequate long-term follow-up.

## 4. GLP-1 Medicines and Cancer Toxicity

It is well known that anticancer therapies are associated with an increased risk of CV disease. In this context, GLP-1 medicines may offer a cardioprotective effect, potentially mitigating treatment-related cardiac risk and improving overall survival. The review identified studies that primarily addressed the impact of GLP-1 medicines among individuals with cancer and their effects on cancer treatment toxicity, primarily, though not exclusively, affecting the heart. Five large real-world studies [49,50,51,52,53] published in 2025 and 2026 reported on the impact of GLP-1 medicines on cardiac and other organ dysfunction among patients with obesity who received treatment for cancer. Specifically, cancer survivors (several cancer types) with T2DM using GLP-1 medicines, compared with non-users, had a 54% lower risk (HR = 0.46, 95% CI: 0.32–0.67, *p* < 0.001) of Major Adverse CV Events (MACE) [49]. Although the study used propensity score matching, residual confounding may still be present, as the univariate analysis may not have accounted for all potentially important variables [49]. Another study examined CV disease among women with breast cancer and T2DM and found a 20% lower risk among GLP-1 medicine users compared to non-users (RR = 0.80, 95% CI: 0.75–0.85, *p* < 0.001) [50]. Importantly, Black or African American women comprised approximately 20% of participants in both study arms, providing greater racial representation that is often not seen in similar studies. In the third study, cancer survivors with diagnosed anthracycline-induced cancer therapy-related cardiac dysfunction (CTRCD) found that GLP-1 medicine users had a 39% lower risk of acute heart failure HR = 0.61, 95% CI: 0.41–0.82, *p* = 0.007) and 42% lower risk of acute renal failure (HR = 0.58, 95% CI: 0.43–0.88, *p* = 0.002), significantly reducing cancer-related adverse clinical outcomes [51]. Similar findings were reported in a study involving patients with a prior cancer diagnosis who developed CTRCD following treatment with cardiotoxic antineoplastic therapies. GLP-1 medicine use was associated with a 31% significantly lower risk of acute heart failure exacerbations compared with non-use (HR = 0.69, 95% CI: 0.56–0.85, *p* < 0.001) [52]. However, the study included patients with a variety of cancer types and comorbid conditions (e.g., T2DM, hypertension) and lacked detailed oncology treatment data. These factors may have introduced heterogeneity and residual confounding, potentially biasing the results. The fifth study evaluated the incidence of MACE among patients with solid tumors and cardiometabolic conditions, including individuals with and without T2DM, who were treated with tirzepatide or other GLP-1 medicines [53]. Compared with GLP-1 medicine users, individuals receiving tirzepatide had a 24% lower risk of MACE (HR = 0.76, 95% CI: 0.62–0.94, *p* = 0.011) [53]. Additionally, tirzepatide users experienced significantly greater reductions in BMI than users of other GLP-1 medicine agents. Proposed mechanisms by which GLP-1 medications may reduce CVD risk include deceased vascular inflammatory signaling, improved arterial function, and reduced oxidative stress [54]. A longer intervention is required to fully understand the impact of GLP-1 medicine on cancer-related outcomes, since the development of some types of cancers usually requires decades of slow cellular growth and genetic mutations [55]. Collectively, these studies suggest that GLP-1 medicines may confer cardioprotective benefits among cancer survivors with T2DM. However, prospective long-term studies and randomized controlled trials are needed to confirm these findings and to better understand the effects of GLP-1 medicines on CV disease outcomes in cancer populations. Table 1 highlights the GLP-1 medicine and cancer studies included in this review. Based on the studies identified for review in Figure 1, Table 1 describes if the study addresses individuals receiving active treatment for cancer, those who have completed treatment, or both.

Cancer side effects can increase in individuals who also take GLP-1 medicines. As previously mentioned, because GLP-1 medicines delay gastric emptying, there has been concern that they may decrease overall oral drug exposure. It appears that GLP-1 medicines can increase, decrease, or have no effect on overall drug exposure [35]. The authors concluded that dose adjustments are probably not required for simultaneous use of GLP-1 medicines and oral chemotherapeutic agents, although this may not apply to individuals with kidney dysfunction or when using chemotherapeutic agents with a narrow therapeutic index [35]. Oral tyrosine kinase inhibitors are subject to high interpatient variability, in part due to drug–drug interactions. As such, GLP-1 medicines may alter a patient’s response to tyrosine kinase inhibitor therapy [56]. Financial toxicity is a reality for many cancer patients [57]. GLP-1 medicines are expensive and not always covered by insurance. For patients without insurance, the costs are greater, leading to socioeconomic inequalities in the healthcare system.

## 5. Patients Receiving GLP-1 Medicines and Anticancer Therapies

Nutritional status is influenced by reduced caloric intake, tumor burden, and medical and surgical anticancer treatments [58], which may cause taste changes, nausea, and/or vomiting. As a result, many cancer survivors are at risk for nutritional deficiencies, increasing the risk of cachexia, protein-energy malnutrition, and/or sarcopenic obesity. These risks may be compounded by GLP-1 medicine therapy. This is especially true for survivors who are undergoing active treatment for their malignancy. Caution before using GLP-1 medicines is especially advised during active treatment, with frequent monitoring of possible sarcopenia, muscle loss, and an unexpected degree of weight loss.

Cancer cachexia is a multifactorial syndrome defined by an ongoing loss of skeletal muscle mass (with or without loss of fat mass) that cannot be fully reversed by conventional nutritional support and leads to progressive functional impairment [59]. The risk of cachexia progression often depends on cancer type and stage, the presence of systemic inflammation, low food intake, inactivity, and lack of response to anticancer therapy [60]. Cancer cachexia is uncommon to rare in early-stage cancer, but common among individuals with advanced cancer [61]. As such, GLP-1 medicine pharmacotherapy should be instituted with caution among individuals with advanced cancer. Long-term management of obesity, including among individuals with cancer, is best managed by a multidisciplinary team of obesity specialists, including dietitians and physicians. Long-term weight management goals should be established before selecting the optimal strategy to achieve the desired weight loss and maintenance of weight loss. If pharmacotherapy is included in the treatment strategy, lifestyle modifications, such as healthy eating and physical activity, should be emphasized to support the long-term maintenance of these behaviors. Two recently published studies demonstrated that continued treatment with tizepatide [62] or substitution of the oral GLP-1 medicine orforglipron after 1 year of semaglitide treatment [63] was more effective for weight maintenance than placebo.

Another concern among cancer patients is the risk of protein-energy malnutrition. One study found that 43% of cancer survivors were at risk for protein-energy malnutrition during their initial medical oncology visit, and the severity was positively associated with advanced cancer stage [64]. Similarly, users of GLP-1 medicines may be at increased risk for protein-energy malnutrition since these medications suppress appetite and have adverse gastrointestinal effects contributing to reduced energy intake and nutritional deficiencies [65]. In addition, one study reported that individuals using GLP-1 medicines consumed insufficient amounts of essential nutrients and had suboptimal protein intake during weight loss [66], thereby increasing their risk of protein-energy malnutrition. Recognizing these concerns, patients should undergo a comprehensive nutritional assessment prior to initiating GLP-1 medicines to identify existing nutritional risks and facilitate the prompt implementation of appropriate nutrition and metabolic support [60]. Despite these recommendations, the prevalence and risk of protein-energy malnutrition among cancer survivors receiving GLP-1 medicines have not been systematically evaluated among individuals receiving both GLP-1 medicines and anticancer therapies.

As already mentioned, some patients with obesity may also be at increased risk of sarcopenic obesity. Individuals with sarcopenic obesity have high adiposity and decreased muscle mass. This is associated with increased lipid accumulation in muscle fibers, leading to a pro-inflammatory state and insulin resistance, both of which are associated with cancer [67]. One study reported that the prevalence of sarcopenic obesity among newly diagnosed cancer survivors was approximately 40–50% [68], a condition associated with lower overall survival and poorer quality of life [69]. Sarcopenic obesity is also associated with a higher risk of dose-limiting toxicity in patients receiving systemic therapy for cancer [70]. For these individuals, it is recommended to not restrict calories, supplement protein intake and increase resistance exercise.

Due to its appetite-suppressing effects, GLP-1 medicine therapy may also increase the risk of nutritional deficiencies. A recent review of observational cohort studies and case reports found that individuals using GLP-1 medicines consumed less than the recommended intake of calcium, iron, vitamin D, and B-complex vitamins [65], potentially increasing risk of low bone mineral density or osteopenia. Bone loss is likely driven by rapid and substantial weight loss [71] and decreased dietary intake associated with GLP-1 medicine use, compounded by cancer- and treatment-related factors that negatively affect bone health [72]. Although GLP-1 medicines may exert beneficial effects on bone metabolism [73], cancer survivors remain particularly vulnerable. For this reason, comprehensive nutritional assessment is essential for health, well-being, and survival in patients undergoing two simultaneous treatments—anticancer therapy and GLP-1 medicines—which may have compounding effects that increase the risk of protein-energy malnutrition, nutritional deficiencies, and adverse outcomes. Table 2 highlights several adverse effects associated with GLP-1 medicines that overlap with those observed during anticancer therapy. These shared risks may contribute to nutritional compromise, increasing risk for cachexia, protein-energy malnutrition, sarcopenia, and reduced treatment tolerance among cancer survivors.

An advisory committee on nutritional priorities to support GLP-1 medicine therapy recommended that treatment be patient-centered, focusing on the patient’s preferences, values, and medical conditions, with monitoring for gastrointestinal side effects prior to initiation of GLP-1 medicine therapy [60]. Additionally, the assessment of muscle function and strength, body composition, and lifestyle behaviors should be part of the clinical evaluation.

There are specific considerations for cancer survivors, which is why it is very important to discuss treatment with the care team. Many clinicians who treat pancreatic cancer survivors avoid GLP-1 medicines because these patients are already at high risk for pancreatitis, and the drugs may further increase that risk [74]. Clinicians are cautious about using GLP-1 medicines during active cancer therapy, especially in patients experiencing side effects such as nausea and vomiting, as GLP-1 medicines may exacerbate these symptoms. These medications often cause dramatic weight loss, including loss of non-obese muscle mass. As such, clinicians need to carefully monitor GLP-1 medicine use in patients undergoing active chemotherapy, as cachexia and sarcopenia can reduce treatment tolerance. Efforts should be made to minimize loss of fat-free mass and skeletal muscle while ensuring adequate protein and nutrient intake and regular physical activity [75]. The safety of GLP-1 medicines, particularly regarding their potential risk of cancer recurrence and survival outcomes, remains an important area of concern. Although preliminary research suggests these agents do not increase the risk of cancer recurrence and may improve survival [76] among several obesity-related cancers, another study reported elevated GLP-1 medicine expression in several malignancies, including cervical squamous cell carcinoma, lung squamous cell carcinoma, stomach adenocarcinoma, and uterine corpus endometrial carcinoma [77], which may be associated with poorer survival outcomes. Further research is warranted to elucidate the mechanisms by which GLP-1 medicines influence cancer biology and disease progression.

## 6. Conclusions

Obesity significantly impacts cancer treatment and survivorship, prompting growing interest in the integration of GLP-1 medicines into oncology care. However, the effects of these agents on survivors actively undergoing cancer treatment as well as long-time survivors remains insufficiently understood. Most of the available evidence is derived from retrospective cohort studies, which are subject to confounding and bias. As such, these findings provide only a partial understanding of how these medications affect survivors, whether or not they are actively receiving treatment. This study had several limitations, including the fact that we only used PubMed/MEDLINE as the search tool for our narrative review. It is possible that other studies may have been found had we searched other databases, which could have filled the gap for the reduced statistical power for subgroup analysis and the absence of ICD-10 coding and staging data. Another important limitation was the lack of information on treatment intent (curative vs. palliative) and timing of systemic cancer therapies (adjuvant vs. neoadjuvant).

Existing data suggest that GLP-1 medicines often decrease body weight in overweight and obese individuals, with outcomes varying by medication, length of use, cancer type, whether the patient is under active treatment, and if so, the specific anticancer therapies administered. Beyond their effects on weight, GLP-1 medicines may also exert pleiotropic benefits, including the potential to reduce cancer treatment-related toxicities, such as cardiotoxicity. The biologic mechanisms underlying these potential benefits are an area of active investigation. However, each cancer type presents potentially unique considerations regarding the use of these agents, depending on the likelihood of weight gain or loss, the specific treatment based on disease type and stage, as well as individual tolerance to these agents during and after treatment. Therefore, individualizing care for the cancer survivor is required. Clinical trials of GLP-1 medicines present unique challenges, including high participant attrition, difficulties with blinding and placebo control, controlling for multiple variables including body weight and presence or absence of T2DM, and the possibility of biased recruitment given the number of individuals already using these agents. To move forward, researchers must prioritize prospective studies, including prospective cohort studies and randomized controlled trials. These studies should evaluate not only weight loss but also meaningful cancer-related outcomes, to determine whether GLP-1 medicines directly influence cancer outcomes. It is critical to document and differentiate intentional vs. unintentional weight loss, as the latter is associated with worse outcomes. Since nutritional considerations after cancer treatment vary by disease site, the studies will best be targeted to the specific site and disease stage (early vs. late) under investigation. There are active investigations on the website clinicaltrials.gov assessing the possible role of GLP-1 medicines in patients with cancer. NCT06877572 seeks to understand how oncologists can help endometrial cancer survivors with obesity to engage in a weight management program and potentially start a GLP-1 medicine. NCT07228741 will determine if tirzepatide and resistance exercise will improve cardiometabolic health in adult survivors of acute lymphoblastic leukemia with obesity. NCT07257484 evaluates the role of tirzepatide in postmenopausal hormone-receptor-positive breast cancer survivors. While we await results from several ongoing RCTs assessing GLP-1 medicine therapy among cancer survivors, adopting a patient-centered approach can include assessment of nutritional status, body composition, treatment tolerability, and quality of life. As with other diseases, personalizing recommendations for the individual cancer survivor should be the goal, with ongoing communication between the individual and their healthcare providers.

## Figures and Tables

**Table 1:** Summary of GLP-1 medicine and cancer studies.

Publication	Study Design	Sample Size	Cancer Type	Cancer Survivor Status +	GLP-1 Medicine	Outcome
[40]	Retrospective Cohort	75 GLP-1 medicine users	Breast Cancer	AT	semaglutide, dulaglutide, tirzepatide, liraglutide, exenatide	Wt loss/mgt
[41]	Retrospective Cohort	1022 GLP-1 medicine users	Breast Cancer	AT	semaglutide, dulaglutide, tirzepatide, liraglutide, exenatide, lixisenatide, albiglutide	Wt loss/mgt
[42]	Retrospective Cohort	1983 GLP-1 medicine users vs. 1983 non-users	Colon Cancer	LTS	GLP-1 medicines (not specified)	Overall mortality
[43]	Retrospective Cohort	103 GLP-1 medicine users vs. 1605 non-users	Colon Cancer	Both	dulaglutide, exenatide, liraglutide, lixisenatide, semaglutide, tirzepatide	5-year mortality
[44]	Retrospective Cohort	751 GLP-1RA users vs. 751 non-users	Colorectal cancer	Both	GLP-1 medicines (not specified)	Overall survival
[45]	Retrospective Cohort	3043 GLP-1 medicine users vs. 3043 non-users	Neuroendocrine tumors	Both	tirzepatide or another GLP-1 medicine	Overall survival
[46]	Retrospective Cohort	3747 GLP-1 medicine users vs. 52,061 non-users	Multiple cancers	AT	semaglutide, dulaglutide, tirzepatide, liraglutide, exenatide	Mortality
[47]	Retrospective Cohort	1023 GLP-1 medicine users vs. 1023 non-users	Ovarian Cancer	AT	semaglutide, dulaglutide, tirzepatide, liraglutide	All-cause mortality, overall survival
[49]	Retrospective Cohort	469 GLP-1 medicine users vs. 469 non-users	Multiple cancers	Both	lixisenatide, semaglutide, exenatide, albiglutide, liraglutide, dulaglutide, or tirzepatide	Major adverse cardiovascular events (MACE)
[50]	Retrospective Cohort	19,608 GLP-1 medicine users vs. 19,608 non-users	Breast cancer	Both	exenatide, liraglutide, dulaglutide, semaglutide albiglutide tirzepatide	MACE
[51]	Retrospective Cohort	201 GLP-1 medicine users vs. 201 non-users	Multiple cancers	LTS	semaglutide, dulaglutide, tirzepatide, or liraglutide	Heart failure, renal function
[52]	Retrospective Cohort	837 GLP-1 medicine users vs. 837 non-users	Multiple cancers	Both	GLP-1 medicines (not specified)	Heart failure exacerbations
[53]	Retrospective Cohort	42,584 GLP-1 medicine users	Multiple cancers	Both	tirzepatide vs. other GLP-1 medicine	MACE

+: Active treatment (AT), Long-term survivor (LTS), or both groups (Both); Weight (wt); management (mgt).

**Table 2:** Risk of GLP-1 medicines and anticancer therapies among cancer survivors.

GLP-1 Risk	Anticancer Therapy Side Effects
Rapid weight loss	Rapid weight loss
Gastrointestinal challenges (nausea, vomiting)	Gastrointestinal challenges (nausea, vomiting)
Nutritional deficiencies, cachexia, protein-energy malnutrition	Nutritional deficiencies, cachexia, protein-energy malnutrition
Sarcopenic obesity	Sarcopenic obesity
Bone loss	Bone loss

## Data Availability

The original contributions presented in this study are included in the article. Further inquiries can be directed to the corresponding authors.
